# Assessing measures of comorbidity and functional status for risk adjustment to compare hospital performance for colorectal cancer surgery: a retrospective data-linkage study

**DOI:** 10.1186/s12911-015-0175-1

**Published:** 2015-07-15

**Authors:** Timothy A Dobbins, Tim Badgery-Parker, David C Currow, Jane M Young

**Affiliations:** Cancer Epidemiology and Services Research, Sydney School of Public Health, University of Sydney, Sydney, NSW Australia; National Centre for Epidemiology and Population Health, Research School of Population Health, Australian National University, Canberra, ACT Australia; Cancer Institute NSW, Sydney, NSW Australia; Surgical Outcomes Research Centre (SOuRCe), Sydney Local Health District, Royal Prince Alfred Hospital, Sydney, NSW Australia

**Keywords:** Risk adjustment, Comorbidity, Surgical outcomes, Administrative data, Charlson comorbidity index, ECOG performance status, ASA score

## Abstract

**Background:**

Comparing outcomes between hospitals requires consideration of patient factors that could account for any observed differences. Adjusting for comorbid conditions is common when studying outcomes following cancer surgery, and a commonly used measure is the Charlson comorbidity index. Other measures of patient health include the ECOG performance status and the ASA physical status score. This study aimed to ascertain how frequently ECOG and ASA scores are recorded in population-based administrative data collections in New South Wales, Australia and to assess the contribution each makes in addition to the Charlson comorbidity index in risk adjustment models for comparative assessment of colorectal cancer surgery outcomes between hospitals.

**Methods:**

We used linked administrative data to identify 6964 patients receiving surgery for colorectal cancer in 2007 and 2008. We summarised the frequency of missing data for Charlson comorbidity index, ECOG and ASA scores, and compared patient characteristics between those with and without these measures. The performance of ASA and ECOG in risk adjustment models that also included Charlson index was assessed for three binary outcomes: 12-month mortality, extended length of stay and 28-day readmission. Patient outcomes were compared between hospital peer groups using multilevel logistic regression analysis.

**Results:**

The Charlson comorbidity index could be derived for all patients, ASA score was recorded for 78 % of patients and ECOG performance status recorded for only 24 % of eligible patients. Including ASA or ECOG improved the predictive ability of models, but there was no consistently best combination. The addition of ASA or ECOG did not substantially change parameter estimates for hospital peer group after adjusting for Charlson comorbidity index.

**Conclusions:**

While predictive ability of regression models is maximised by inclusion of one or both of ASA score and ECOG performance status, there is little to be gained by adding ASA or ECOG to models containing the Charlson comorbidity index to address confounding. The Charlson comorbidity index has good performance and is an appropriate measure to use in risk adjustment to compare outcomes between hospitals.

**Electronic supplementary material:**

The online version of this article (doi:10.1186/s12911-015-0175-1) contains supplementary material, which is available to authorized users.

## Background

Comparing outcomes between clinicians, institutions or area health services requires consideration of potential confounding factors, such as patient age, health status or disease severity, that could account for any observed differences in outcomes. Risk adjustment is a statistical technique used to account for variations in outcome that arise from differences in the characteristics of patients, so as to provide a fair and more accurate comparison of health services. Patient age, sex, cancer site and cancer stage are commonly used in risk adjustment models as they have high levels of completeness and accuracy in routinely collected datasets such as cancer registries and hospital episode collections.

Adjustment for comorbid conditions is common when studying outcomes following cancer surgery [1]. The Charlson comorbidity index summarises information about the presence of a number of medical conditions, assigns a severity score to each, and sums the severity scores to create a single measure of comorbidity [2]. Algorithms have been derived to calculate the Charlson comorbidity index from hospitalisation records coded using the Ninth [3, 4] and Tenth [5, 6] Revisions of the International Classification of Diseases. The Charlson comorbidity index has been shown to be a good predictor of both short-term (in hospital and 30-days) and longer-term (12-month) mortality [7].

Functional status is an additional patient factor that may explain differences in outcome. One measure of functional status is the Eastern Cooperative Oncology Group (ECOG) Performance Status, [8] a score ranging from 0 (“fully active”) through 3 (”capable of only limited self-care”) to 5 (“dead”). An alternative measure of a patient’s general health is the American Society of Anesthesiologists (ASA) score [9]. This measure of physical status ranges from 1 for a normal healthy patient to 5 for a moribund patient who is not expected to survive surgery and is recorded routinely by anaesthetists prior to any surgical procedure. ECOG and ASA scores are available in some administrative data collections.

While recent studies have assessed the predictive capacity of comorbidity and health status measures in risk adjustment for outcomes of cancer surgery, [10, 11] they did not consider performance status. A study that compared comorbidity index, ASA and ECOG performance status [12] considered each measure separately, and did not assess the additional effect of health and performance status over comorbidity. In a recent study comparing ASA with ECOG, [13] no comparison was made with comorbidity index as it was not possible to derive a comorbidity index from the available data. Additionally, these studies used data extracted from patients’ medical records, not routinely collected data.

Therefore, we conducted this study to assess how frequently the ASA score and ECOG performance status can be obtained from population-based administrative data collections in New South Wales, the most populous state in Australia, and to assess the contributions of ASA score and ECOG performance status additional to the Charlson comorbidity index, in comparing outcomes of colorectal cancer surgery between hospital peer groups based on risk adjustment models.

## Method

### Data sources

Our study population comprised all people with an incident cancer of the colon or rectum registered in NSW, the most populous state in Australia. We used the first recorded tumour of the colon or recto-sigmoid junction (ICD-O-3 codes: C18, C19) or rectum (C20) registered on the NSW Central Cancer Registry (CCR) between 1 January 2000 and 31 December 2008. The NSW CCR, a population-based register of all cancers in NSW residents, receives notifications from public and private hospitals, departments of radiation oncology, nursing homes, pathology laboratories, outpatient departments and day procedure centres as a statutory requirement.

Cancer notifications were linked to the Admitted Patients’ Data Collection (APDC), the Clinical Cancer Registry (ClinCR) and the NSW Registry of Births, Deaths and Marriages (RBDM). The APDC contains demographic and episode-related information on all hospital separations for public and private hospitals in NSW, [14] while the ClinCR contains clinically-based information, including the ECOG performance status, for patients with cancer treated in public facilities within six area health services [15]. Hospital admissions with procedures defined as being for curative intent were used, [15] with these procedures based on clinical consultation and previously used procedure codes [16, 17]. Where patients had more than one hospital admission with a procedure of interest, the first admission was used in analysis. We used hospital records with separations in 2007 and 2008, as this was the period for which clinical cancer registry data were available. Fact of death was obtained from the RBDM for the period up to 2010.

Probabilistic data linkage was conducted by the NSW Centre for Health Record Linkage, and we received only anonymised data. Ethics approval for the study was given by the NSW Population and Health Services Research Ethics Committee (HREC/11/CIPHS/32).

### Outcomes

We considered three binary outcomes. Mortality was assessed at 12 months, measured from date of procedure until death from any cause. Hospital stay following surgery was measured from the date of procedure until separation, accounting for transfers, and categorized based on exceeding the recommended length of stay for procedures based on the Australian Refined Diagnostic Related Group [18]. This allowed for length of stay to be assessed based on procedure complexity. Readmission within 28 days was derived from the date of separation until any subsequent readmission.

### Peer group

Peer groups classify similar hospitals based on the number of separations, primary role and location (metropolitan or rural). Admitting hospitals were classified into one of six groups based on NSW Peer Group definitions. [19].

### Measures of patient health

We calculated the Charlson comorbidity index using codes for comorbid conditions recorded during the index admission in the APDC. We excluded metastatic cancer from the calculation as in the context of cancer treatment, this is part of the condition of interest rather than a comorbidity. Charlson comorbidity indices were categorised into values of 0, 1 and 2 or more in risk adjustment models as very few patients (8 %) had scores of two or higher.

The ASA score was obtained from the suffix of ICD procedure codes indicating administration of an anaesthetic (see Additional File 1). Possible ASA scores are 1 (normal healthy patient); 2 (mild systemic disease); 3 (severe systemic disease); 4 (severe systemic disease that is a constant threat to life) or 5 (moribund patient who is not expected to survive) [9]. We grouped ASA scores of 4 or 5 in risk adjustment models due to the sparseness of higher scores.

ECOG performance status was obtained from ClinCR records. We used the ECOG performance status recorded closest in time on or before the data of procedure. Possible ECOG scores are 0 (fully active, no performance restriction); 1 (restricted in physically strenuous activity but ambulatory, able to carry out work of a light or sedentary nature); 2 (ambulatory and capable of all self-care but unable to carry out any work activities. Up and about more than 50 % of waking hours); 3 (capable of only limited self-care, confined to bed or chair more than 50 % of waking hours); 4 (Completely disabled, cannot carry out any self-care. Totally confined to bed or chair) or 5 (dead) [8]. We grouped ECOG performance status of 2 or higher in risk adjustment models due to the sparseness of higher scores.

### Other patient factors

Extent of tumour spread, obtained from the CCR, summarises the most aggressive extent of the disease based on diagnostic and therapeutic evidence within four months of diagnosis [20]. Patient sex, age and emergency status of admission were obtained from the APDC, with age calculated at date of procedure. Country of birth, also obtained from APDC, was classified as Australian versus other.

### Statistical analysis

Descriptive statistics were used to compare patient characteristics for patients with non-missing values of Charlson comorbidity index, ASA score and ECOG performance status. Associations between the three patient health measures were summarised using Spearman’s rank correlation coefficients.

Risk adjustment models were constructed using two-level multilevel logistic regression models to compare the binary outcome measures across the peer groups, with patients (level-1) clustered within hospitals (level-2). Random-intercept models were fitted, with hospital included as a random effect. Only patients with non-missing values of Charlson comorbidity index, ASA score and ECOG performance status were used so that risk adjustment models could be compared on the same group of patients. Hence we used only records from patients treated in public hospitals. As such, only the four peer groups representing public hospitals were considered: Principal Referral A, Principal Referral B, Major Metropolitan and Major Non-Metropolitan. Major Metropolitan and Non-Metropolitan peer groups were grouped together due to the low patient numbers in Major Non-Metropolitan hospitals.

We evaluated the utility of ASA score and ECOG performance status by fitting all combinations of these over and above a base model assessing the association between hospital peer group and each of the three study outcomes (12-month mortality, extended length of stay and readmission within 28 days) adjusted for patient age, sex, extent of disease, emergency status of admission and Charlson comorbidity index. Model performance was assessed using Akaike’s information criterion (AIC), with lower values indicating a better fitting model. We also calculated the *C* statistic, summarising the concordance between observed and predicted events. Concordance ranges between 0.5 (a model with no predictive ability) and 1.0 (a model with perfect predictive ability) [21]. Finally, we compared estimated logistic regression parameters for hospital type to assess the extent of confounding explained by the inclusion of ASA score and ECOG performance status.

Data processing and descriptive analyses were conducted using Version 9.3 of the SAS System for Windows (Cary, NC, USA), with multilevel logistic modelling conducted using adaptive quadrature via the xtmelogit command of Stata Version 12 for Windows (College Station, TX: StataCorp LP).

## Results

We obtained records from 6,964 individuals who were operated in 2007 or 2008, and their characteristics are presented in Table 1. The Charlson comorbidity index could be calculated for the entire sample, as it is based on comorbid conditions recorded during the index admission. ASA score was recorded for 78.0 % of these individuals, and while they were similar to those without ASA score recorded for most characteristics, those with ASA score recorded were more likely to have been operated on in a public hospital.

**Table 1:** Characteristics of patients, operated on between 2007 and 2008 for cancer of the colon or rectum in NSW, Australia

		Full cohort (*n* = 6964)			Patients operated on between 2007 and 2008 in a public hospital with a ClinCR record 01/2007 to 12/2008 (*n* = 3,079)	
		Charlson recorded (*n* = 6,964, 100 %)	ASA Status recorded (*n* = 5430; 78.0 %)	ASA Status missing (*n* = 1534; 22.0 %)	ECOG-PS recorded (*n* = 748; 24.3 %)	ECOG-PS missing (*n* = 2,331; 75.7 %)
Age at admission	Mean (range)	68.9 (14 to 99)	69.0 (14 to 99)	68.6 (21 to 97)	68.0 (15 to 95)	69.8 (20 to 97)
	Median (IQR)	70 (61 to 78)	70 (61 to 78)	69 (61 to 78)	69 (60 to 77)	71 (62 to 79)
Sex	Male	3763 (54 %)	2930 (54 %)	833 (54 %)	435 (58 %)	1241 (53 %)
	Female	3201 (46 %)	2500 (46 %)	701 (46 %)	313 (42 %)	1090 (47 %)
Emergency^a^	Non-emergency	5519 (89 %)	4353 (89 %)	1166 (91 %)	607 (86 %)	1906 (83 %)
	Emergency	659 (11 %)	539 (11 %)	120 (9 %)	101 (14 %)	404 (17 %)
Country of birth^a^	Non-Australian	1920 (28 %)	1519 (28 %)	401 (26 %)	338 (45 %)	816 (35 %)
	Australian	4997 (72 %)	3876 (72 %)	1121 (74 %)	410 (55 %)	1506 (65 %)
Extent of disease	Localised	2549 (37 %)	1928 (36 %)	621 (40 %)	197 (26 %)	798 (34 %)
	Regional	3218 (46 %)	2544 (47 %)	674 (44 %)	387 (52 %)	1108 (48 %)
	Distant	843 (12 %)	697 (13 %)	146 (10 %)	133 (18 %)	327 (14 %)
	Unknown	354 (5 %)	261 (5 %)	93 (6 %)	31 (4 %)	98 (4 %)
Private hospital	Public	3848 (55 %)	3202 (59 %)	646 (42 %)	748 (100 %)	2331 (100 %)
	Private	3116 (45 %)	2228 (41 %)	888 (58 %)		

^a^Missing values: Emergency status (*n* = 786), Country of birth (*n* = 47)

There were 3,848 individuals who had a potentially curative surgical procedure at a public hospital in 2007 or 2008. Of these, 3,079 individuals had a record in the Clinical Cancer Registry and 748 (24.3 %) had an ECOG performance status recorded before their cancer surgery. Patients with a recorded ECOG performance status were more likely to be male, have a non-Australian country of birth, and have a more advanced extent of disease compared to those without ECOG performance status.

The three measures of patient health status were not highly correlated with one another (*r*_ASA,Charlson_ = 0.29, *r*_ASA,ECOG_ = 0.15, *r*_ECOG,Charlson_ = 0.11) (Table 2).

**Table 2:** Cross tabulations of Charlson, ASA and ECOG scores (*n* = 575)

a)					
ASA score	Charlson score				
	0	1	2+		
1	43	0	2	45	
2	270	6	10	286	
3	166	16	22	204	
4	20	3	11	34	
5	4	0	2	6	
	503	25	47		
b)					
ECOG	Charlson score				
	0	1	2+		
0	246	10	19	275	
1	212	8	14	234	
2	32	5	11	48	
3	13	2	2	17	
4	0	0	1	1	
c)					
ASA score	ECOG				
	0	1	2	3	4
1	25	19	1	0	0
2	152	106	21	7	0
3	82	94	21	7	0
4	13	13	4	3	1
5	3	2	1	0	0

There were 575 records with non-missing measures of health status and other covariates that could be used in risk adjustment comparisons. The majority had Charlson scores of 0 (*n* = 502, 87.5 %) with 25 (4.4 %), 38 (6.6 %), 3 (0.5 %) and 6 (1.0 %) scoring 1,2, 3 and 4 respectively. There were no Charlson scores higher than 4. Results of model performance appear in Table 3. For each outcome, the estimated intracluster correlation coefficient was zero for all models.

**Table 3:** Summaries of multilevel logistic regression model performance for three binary outcomes

	365-day mortality		Extended length of stay		28-day readmission	
	(Event rate: 77/575; 13.4 %)		(Event rate: 92/575; 16.0 %)		(Event rate: 109/575; 19.0 %)	
Model	AIC	C	AIC	C	AIC	C
Base	396.9	0.782	472.0	0.731	572.8	0.606
Base + ASA	392.8	0.797	433.6	0.792	576.5	0.621
Base + ECOG	393.9	0.788	472.5	0.735	566.8	0.631
Base + ASA + ECOG	390.1	0.802	433.9	0.799	570.2	0.643

Base model comprises Charlson comorbidity index, age, sex, extent of cancer disease, emergency presentation and hospital type

Including ASA score and ECOG performance status reduced the AIC for each outcome, but the effect was not consistent. The AIC statistic was minimised by: the inclusion of both measures for 365-day mortality; the inclusion of ASA score for extended length of stay and the inclusion of ECOG performance status for 28-day readmission. Concordance was maximised by the inclusion of both measures for all outcomes. However the maximum concordance for each outcome was similar to that of the best performing models based on AIC statistics.

The effect of including ASA score and ECOG performance status on the logistic regression parameter estimates for hospital-type is summarised in Table 4. Changes in parameter estimates due to adjusting for different measures of health status were small, relative to the parameter estimates’ standard errors, and there was no consistent pattern in the confounding effect of ASA score and ECOG performance status.

**Table 4:** Peer-group parameter estimates from multilevel logistic regression analysis for three binary outcomes

	365-day mortality		Extended length of stay		28-day readmission	
Model	Principal Referral B^a^	Major Metro and Non-Metro^a^	Principal Referral B^a^	Major Metro and Non-Metro^a^	Principal Referral B^a^	Major Metro and Non-Metro^a^
	b (95 % CI)	b (95 % CI)	b (95 % CI)	b (95 % CI)	b (95 % CI)	b (95 % CI)
Base	1.83 (1.12, 2.55)	1.24 (0.60, 1.87)	0.17 (−0.50, 0.84)	0.11 (−0.43, 0.66)	0.27 (−0.34, 0.87)	0.31 (−0.17, 0.79)
Base + ASA	1.87 (1.14, 2.61)	1.32 (0.67, 1.96)	0.08 (−0.65, 0.81)	0.21 (−0.37, 0.79)	0.27 (−0.34, 0.88)	0.33 (−0.15, 0.81)
Base + ECOG	1.67 (0.94, 2.40)	1.24 (0.60, 1.88)	0.03 (−0.67, 0.72)	0.10 (−0.46, 0.65)	0.47 (−0.15, 1.09)	0.31 (−0.17, 0.80)
Base + ASA + ECOG	1.70 (0.95, 2.45)	1.31 (0.66, 1.97)	−0.08 (−0.83, 0.68)	0.20 (−0.39, 0.78)	0.48 (−0.15, 1.10)	0.34 (−0.15, 0.83)

Base model comprises Charlson comorbidity index, age, sex, extent of cancer disease, emergency presentation and hospital type

^a^Relative to Principal Referral

## Discussion

Our study reports on the utility ASA score and ECOG performance status for risk adjustment over and above Charlson comorbidity index using routinely collected, population-based data for NSW. The correlation between the three measures was not strong, suggesting that the measures represent independent attributes of patient well-being. Our study found that, for patients with non-missing Charlson comorbidity index, ASA score and ECOG performance status, there was no clear optimal measure of patient-health for risk adjustment. However, there was no clearly deficient measure. We also found that, after including Charlson comorbidity index, there was little to be gained by adding ASA score or ECOG performance status when comparing outcomes between hospital types.

Within the NSW hospital episode statistics (the APDC), it is standard coding practice that only comorbid conditions which have an impact on the admission of interest are coded. While this means that the Charlson scores derived from the APDC diagnosis codes are based on conditions likely to impact outcome, it leads to under-estimation of the prevalence of comorbid conditions in the patient cohort [22]. We have previously investigated the impact of including additional comorbidity information from previous hospital admissions in risk adjustment models to compare hospital cancer outcomes [13]. This previous work demonstrated that although Charlson scores were higher when more sources of comorbidity information were included, there was little change in the performance of Charlson score in the hospital risk adjustment models. Thus, for comparison of hospital cancer outcomes, calculating the Charlson score from comorbidity information within the index admission was the most efficient approach, and the approach used in this study. We acknowledge however that additional comorbidity information from other sources could improve risk adjustment models in other contexts, for example to compare individual patient outcomes.

While ASA score is reported more commonly in public hospitals than private, patients with ASA score have similar characteristics to those with no recorded ASA score. The reporting of ECOG performance status however, is limited. Only a quarter of patients whose cancer surgery was conducted in a public hospital had an ECOG performance status recorded before their cancer surgery. While we used the ECOG performance measured closest in time prior to cancer surgery, the ideal would be to record ECOG close as possible to surgery, possibly in a preoperative setting. As the analyses in this study were necessarily restricted to a subset of patients with colorectal cancer in NSW who were treated in a public hospital and had complete data for Charlson, ECOG and ASA, it is possible that these findings do not generalise to other patient groups. This should be further investigated in future studies.

The findings from this study regarding predictive ability agree with those from other published work. While comorbidity (as measured by either ASA or Charlson comorbidity index) has been demonstrated as a risk factor for poor outcomes in studies of colorectal cancer surgery, different comorbidity indices added little to the predictive ability regression models, [10] and yielded strikingly similar results [11]. Similar performance of ASA, Charlson comorbidity index and ECOG performance status was seen in a study that modelled90-day post-surgery mortality for carcinoma of the bladder [12]. It should be noted though, that these studies were based on data extracted from primary medical records. Recently, cancer-specific measures of comorbidity (‘C3 indices’) developed using administrative data have been found to have slightly improved performance compared with the Charlson index for colorectal cancer [23]. However, the developers conclude that the differences are so small as to be insignificant, and that any measure of comorbidity will suffice in most circumstances [23]. Consistent with these findings, our study suggests that alternative measures of patient health derived from administrative data collections provide similar predictive ability.

While our study is based on population-level data from high quality data collections, there are a number of limitations. There were only two years of data with the potential for ECOG performance status to be measured, and only a small proportion of patients had ECOG performance status recorded. We could not include patients who received surgery in private hospitals, as ECOG performance status was recorded in the Clinical Cancer Registry which does not yet include private hospitals. Those with non-missing ECOG performance status differed systematically from the entire cohort, and it is not clear whether the same conclusions would be reached in a more representative population. While multiple imputation methods could be used to include patients with missing data, the high proportion and likely non-random nature of the missing ECOG data in our study were against this approach [24]. We could not assess thirty-day mortality, arguably a more important indicator of surgical quality, in the risk adjustment models due to the low numbers of deaths. Finally, the levels of completeness of data fields likely vary in different settings such as the public and private sector, and within and between countries, so our findings may not generalise to other contexts.

## Conclusion

ECOG performance status has some use in risk adjustment, but its routine use in population-based comparisons of hospital outcomes cannot be recommended due to limited coverage. Coverage of ASA scores is greater than for ECOG performance status, but there are still substantial issues of non-reporting, particularly in private hospitals.

While predictive ability of regression models is maximised by inclusion of one or both of ASA score and ECOG performance status, there is little to be gained by adding ASA or ECOG to models containing the Charlson comorbidity index to address confounding. The Charlson comorbidity index is an appropriate measure to use in risk adjustment to compare outcomes between hospitals.

## Additional file

Additional file 1:
**ICD Procedure codes used to obtain ASA score.**

## Abbreviations

ECOG: Eastern Cooperative Oncology Group
ASA: American Society of Anesthesiologists
NSW: New South Wales
CCR: Central Cancer Registry
ClinCR: Clinical Cancer Registry
APDC: Admitted Patients’ Data Collection
RBDM: NSW Registry of Births, Deaths and Marriages
AIC: Akaike Information Criterion
