# Supplementary material for: Assessing measures of comorbidity and functional status for risk adjustment to compare hospital performance for colorectal cancer surgery: a retrospective data-linkage study
Source: BMC Med Inform Decis Mak. 2015 Jul 15;15:55. doi: 10.1186/s12911-015-0175-1 (PMC4502567; doi:10.1186/s12911-015-0175-1)
Supplement: Additional file 1: — ICD Procedure codes used to obtain ASA score. [file 12911_2015_175_MOESM1_ESM.pdf]

ICD Procedure codes used to obtain ASA score

92508: Neuraxial block

92509: Regional block, nerve of head or neck

92510: Regional block, nerve of trunk

92511: Regional block, nerve of upper limb

92512: Regional block, nerve of lower limb

92513: Infiltration of local anaesthetic

92514: General anaesthesia

92515: Sedation

92519: Intravenous regional anaesthesia
